# Acetylation of androgen receptor by ARD1 promotes dissociation from HSP90 complex and prostate tumorigenesis

**DOI:** 10.18632/oncotarget.12163

**Published:** 2016-09-21

**Authors:** John S. DePaolo, Zehua Wang, Jianhui Guo, Guanyi Zhang, Chiping Qian, Haitao Zhang, Jovanny Zabaleta, Wanguo Liu

**Affiliations:** ^1^ Department of Genetics, Louisiana State University Health Sciences Center, New Orleans, LA, 70112, USA; ^2^ Stanley S. Scott Cancer Center, Louisiana State University Health Sciences Center, New Orleans, LA, 70112, USA; ^3^ Department of Pathology, Tulane University School of Medicine, New Orleans, LA, 70112, USA; ^4^ Department of Pediatrics, Louisiana State University Health Sciences Center, New Orleans, LA, 70112, USA

**Keywords:** ARD1, AR acetylation, AR-HSP90 dissociation, prostate tumorigenesis

## Abstract

Prostate cancer is an androgen receptor (AR)-driven disease and post-translational modification of AR is critical for AR activation. We previously reported that Arrest-defective protein 1 (ARD1) is an oncoprotein in prostate cancer. It acetylates and activates AR to promote prostate tumorigenesis. However, the ARD1-targeted residue within AR and the mechanisms of the acetylation event in prostate tumorigenesis remained unknown. In this study, we show that ARD1 acetylates AR at lysine 618 (K618) *in vitro* and *in vivo*. An AR construct with the charged lysine substitution by arginine (AR-618R) reduces RNA Pol II binding, AR transcriptional activity, prostate cancer cell growth, and xenograft tumor formation due to attenuation of AR nuclear translocation, whereas, construct mimicking neutral polar substitution acetylation at K618 by glutamine (AR-618Q) enhanced these effects beyond that of the wild-type AR. Mechanistically, ARD1 forms a ternary complex with AR and HSP90 *in vitro* and *in vivo*. Expression of ARD1 increases levels of AR acetylation and AR-HSP90 dissociation in a dose dependent manner. Moreover, the AR acetylation defective K618R mutant is unable to dissociate from HSP90 while the HSP90-dissociated AR is acetylated following ligand exposure. This work identifies a new mechanism for ligand-induced AR-HSP90 dissociation and AR activation. Targeting ARD1-mediated AR acetylation may be a potent intervention for AR-dependent prostate cancer therapy.

## INTRODUCTION

Androgen receptor (AR) plays a crucial role in prostate cancer (PCa) [1, 2]. Activation of AR via mutation, amplification, overexpression, or posttranslational modification leads to PCa initiation and progression [3–5]. Although androgen deprivation therapy (ADT) is initially effective, the unconfined disease inevitably overcomes the androgen blockade and recurs as lethal castration-resistant PCa (CRPC) [6]. Alternative strategies that inhibit AR activity without contributing to disease progression are needed.

In prostate cells, prior to androgen binding, AR is inactively bound to a foldosome of chaperone proteins within the cytoplasm, including HSP90, preventing nuclear localization while maintaining ligand access [7, 8]. Upon androgen binding, AR undergoes a conformational change leading to AR-HSP90 dissociation, rapid nuclear translocation, and AR target gene transcription [9]. Unchecked, this process eventually leads to prostate tumorigenesis. Disrupted AR and HSP90 interaction via HSP90 inhibition has been utilized as a therapeutic target in PCa clinical trials, but with limited success thus far [10]. New approaches for targeting the interaction of AR and HSP90, as well as the androgen-driven AR nuclear translocation may provide more efficacious therapeutic strategies while avoiding the deleterious effects of ADT.

Posttranslational modification of AR, including acetylation, plays a crucial role for AR activation [11]. To date, several acetyltransferases have been identified to acetylate AR including p300 [12, 13]. P300 acetylates AR at lysines KLKK^633^ and is critical for AR activation, lncoRNA binding, and prostate tumor development [13, 14]. Arrest defective-1 protein (ARD1, also known as Naa10p) is another acetyltransferase [15, 16]. It acetylates amino acids at the N-terminal of protein (N-alpha-acetylation) or lysines within proteins (epsilon-acetylation), both of which play important roles in several types of cancer through acetylating different target proteins [17–21]. Previously, we reported that the level of ARD1 is consistently higher in PCa, and that ARD1 activates AR through ARD1-mediated AR acetylation [22]. Moreover, depletion of ARD1 diminishes LNCaP cell xenograft tumor growth [22]. However, the specific target residue of ARD1-mediated AR acetylation, has not yet been identified. And the bio-pathological roles and mechanisms of AR acetylation in AR activation and prostate tumorigenesis have yet to be elucidated. These include AR and HSP90 interaction and AR nuclear translocation. Inhibition of an AR activator that modulates both AR activity and AR-HSP90 interaction could serve as a powerful, synergistic strategy to suppress PCa progression.

## RESULTS

### ARD1 acetylates AR at K618 *in vitro* and *in vivo*

**Figure 1:**
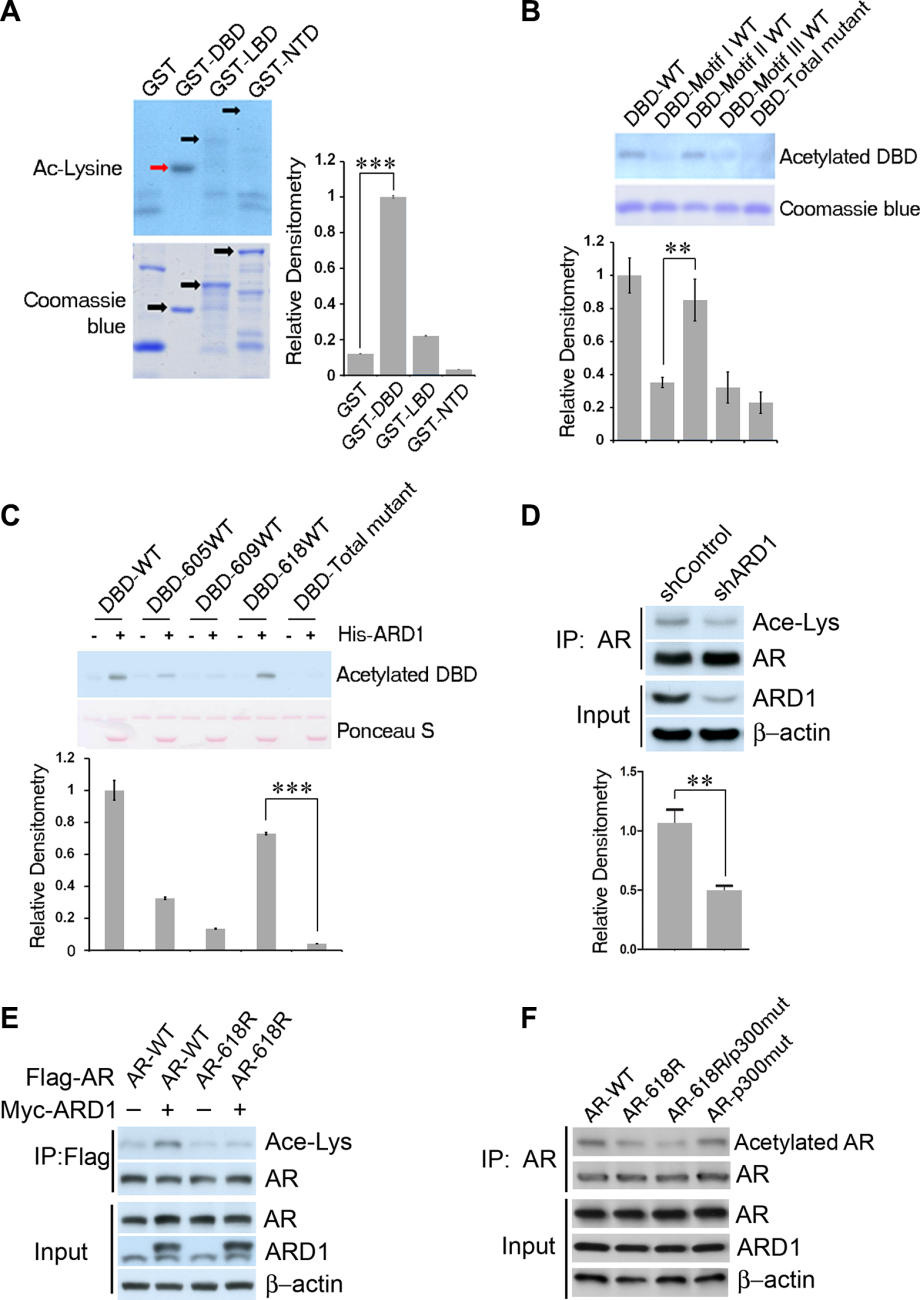
AR is acetylated by ARD1 at K618 *in vivo* and *in vitro* (**A**–**C**) Immunoblots and graphs of normalized relative densitometry of individual GST-AR fragments (A), of individual DBD lysine-containing motif-mutants (B), and of individual DBD single-lysine-WT mutants (C) following *in vitro* acetylation assay and Western blot analyses with anti-acetylated lysine antibody. (**D**) Silencing ARD1 by shRNA inhibits AR acetylation *in vivo*. Cell lysates from LNCaP cells transfected with shARD1 or controls were immunoprecipitated with AR antibody; the level of acetylated AR was measured by Western blotting using the anti-acetylated lysine antibody. (**E**) Overexpression of ARD1 increased acetylation level of WT-AR but not AR-618R mutant *in vivo*. Cell lysates from 293 cells co-transfected ARD1 with WT-AR or AR-618R mutant construct were immunoprecipitated with AR antibody; the level of acetylated AR was measured by anti-acetylated lysine antibody. (**F**) Immunoblot of *in vivo* acetylation of AR-WT, AR-618R, AR-618R/p300mut, or AR-p300mut, respectively. Cell lysates from 293T cells co-transfected with ARD1 and each individual AR construct were immunoprecipitated with AR antibody and acetylation levels were measured with anti-acetylated lysine antibody. All experiments were repeated at least thrice and the data were presented as the mean of the experiments (± SEM). Significance of statistical analysis was indicated as *(*p* < 0.05), **(*p* < 0.01), and ***(*p* < 0.001).

We previously reported that ARD1 acetylates AR but the AR acetylation site(s) targeted by ARD1 is different from that of p300 which targets lysine residues (KLKK^633^) [22]. To determine which lysine residue is acetylated by ARD1, we synthesized GST-tagged AR fragments and subjected them to *in vitro* acetylation assays in the presence of *in vitro* synthesized His-ARD1 and acetyl CoA, respectively (Figure S1). We demonstrated that the DNA binding domain (DBD) of AR is strongly acetylated by ARD1 (Figure 1A). Since DBD comprises three lysine-containing motifs (Figure S2A), we then created three DBD mutant constructs in which all lysines in two motifs were mutated leaving one motif intact, as well as one DBD-total mutant construct in which all lysines in all three motifs were mutated (Figure S2B). The DBD mutants were subjected to *in vitro* acetylation assays again, and motif II was identified as the ARD1 target (Figure 1B). Motif II of the DBD contains three lysines at residues 605, 609, and 618. To determine which lysine in motif II is acetylated by ARD1, we mutated all but one lysine, respectively, within motif II and again subjected each DBD mutant to *in vitro* acetylation assay (Figure S2C). Lysine 618 (K618) was identified as the primary target of ARD1 (Figure 1C). These data suggested that K618 is the ARD1 acetylation target in AR *in vitro*.

To determine if K618 is the ARD1-acetylated site *in vivo*, we first silenced ARD1 by using shRNA in LNCaP and showed that the acetylation levels of the endogenous AR in these cells are greatly reduced (Figure 1D). Next, we transfected wild-type AR-K618 or mutant AR-618R (in which the lysine was replaced by arginine) expression construct in 293 cells and examined the acetylation level of AR-K618 or AR-618R using an acetylation-specific antibody. The result demonstrated that the absence of the K618 site severely reduced the acetylation level of AR by over 90 percent *in vivo* (Figure 1E). Since KLKK^633^ in AR are the acetylation target for p300/Tip60, we transfected mutant AR constructs in which either KLKK^633^ or both KLKK^633^ and K618 were replaced by arginine to compare the levels of *in vivo* acetylation. The results indicated that absence of K618 severely limited acetylation, and the absence of both K618 and K630/632/633 nearly abolished AR acetylation (Figure 1F). Notably, loss of the ARD1 target site had a much more negative impact on AR acetylation level than the loss of the p300/Tip60 sites. Taken together, these data suggest that K618 is the primary ARD1-specific acetylation target within AR *in vivo* and *in vitro*.

### ARD1-mediated acetylation enhances AR activity

**Figure 2:**
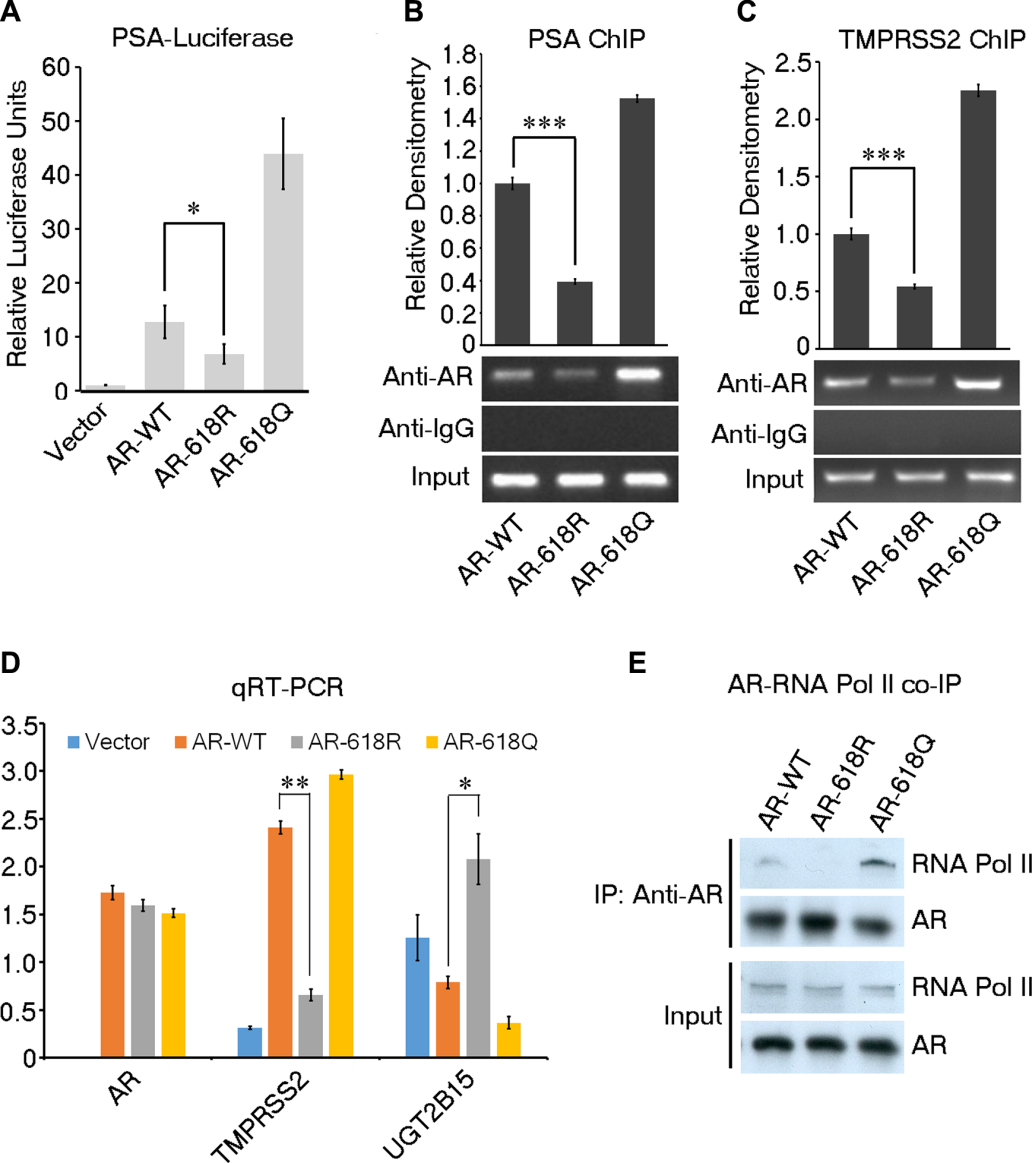
ARD1-dependent acetylation enhances AR transcriptional activity (**A**) Luciferase reporter assays in DU 145 cells co-transfected with PSA-Luc reporter with AR-WT, AR-618R, AR-618Q, or empty vector respectively. Results presented as mean of three replicates ± SD. (**B** and **C**) Differential binding of AR-WT, -618R, or -618Q to PSA and TMPRSS2 promoters by ChIP assays using anti-AR antibodies. Normalized relative densitometry was shown above. (**D**) Quantification of *in vivo* expression levels of mRNA for TMPRSS2 (AR positively-regulated) and UGT2B15 (AR negatively-regulated) in DU 145 cells stably expressing AR-WT, AR-618R, AR-618Q, or empty vector by qRT-PCR. Values represent the mean of triplicate experiments ± SD. (**E**) Immunoblot of co-immunoprecipitated RNA polymerase II by transiently-transfected AR-WT, AR-618R, or AR-618Q in DU 145 cells.

Next, we investigated the impact of ARD1-mediated acetylation on the activity of AR. We created an AR-618R mutant construct to abolish ARD1-mediated acetylation and an AR-618Q mutant construct to mimic constitutive acetylation. Using AR-negative DU 145 PCa cells, we analyzed the impact of WT-AR-K618, mutants AR-618R and AR-618Q on PSA-luciferase reporter activity. We showed that AR-618Q enhanced the promoter activity, but AR-618R significantly diminished the activity (Figure 2A). In agreement, chromatin immunoprecipitation (ChIP) analysis showed that expression of AR-618Q enhanced its interaction with the PSA or TMPRSS2 promoters as compared to WT-AR-K618, while AR-618R significantly reduced promoter binding (Figure 2B, 2C). DU 145 cells stably expressing each of the three AR constructs were created. RNA from each of the three stable cell pools was harvested and subjected to qRT-PCR analysis. While AR-618Q enhanced AR target gene transcription as compared to WT-ARK618, the presence of AR-618R inhibited it (Figure 2D). In addition, AR-618Q co-immunoprecipitated more readily with RNA polymerase II as compared to WT-AR-K618, while the interaction of AR-618R with RNA polymerase II was greatly reduced (Figure 2E). These data demonstrated that ARD1-specific acetylation at lysine 618 enhances transcriptional activity of AR.

### ARD1-mediated AR acetylation enhances prostate tumorigenesis

**Figure 3:**
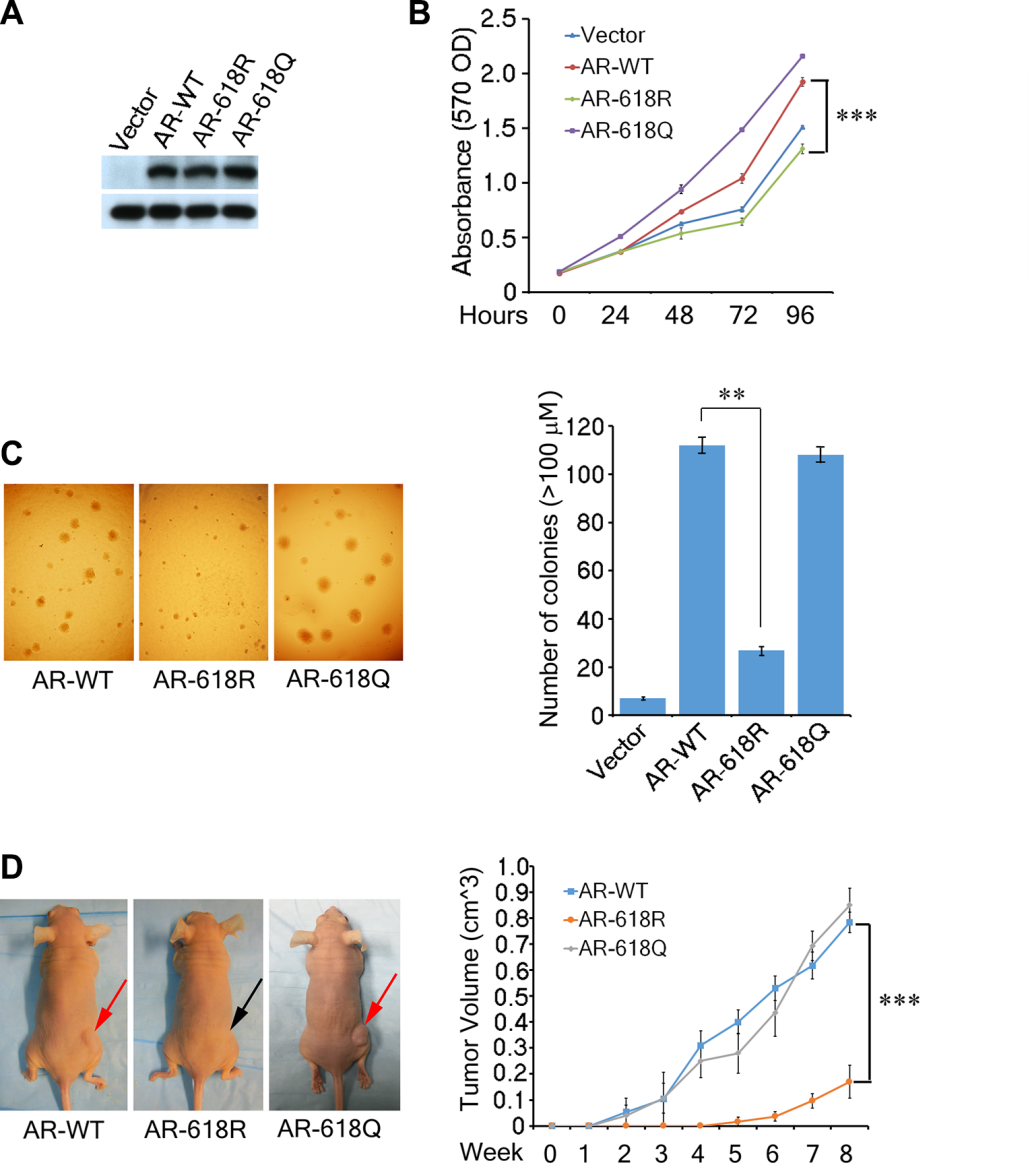
AR Acetylation at K618 enhances prostate cell oncogenecity and xenograft tumor growth (**A, B**) The growth curves of DU 145 cell pools stably-expressing AR-WT, -618R, -618Q, or empty vector analyzed by MTT assays over a 5-day time course. The data were presented as the mean of triplicate experiments (± SEM). Stable expression levels of AR are shown by Western blot (left). (**C**) Anchorage-independent colony formation of the same DU 145 stable cells were assayed by seeding 3,000 cells in soft agar and incubated for 21 days. Colonies (> 100 μm) were counted in each plate and presented graphically (right). (**D**) Tumor volumes of the DU 145 cells stably expressing each AR species in male nude mice measured weekly. The graphed results represent mean tumor volume ± SEM (*n* = 3 mice/group). All of these cells were grown in RPMI phenol red-free 1640 media with 10% charcoal stripped FBS and the addition of R1881 to 1 nM.

To understand the pathological impact of ARD1-mediated AR acetylation, we measured its tumorigenic potential using pools of DU 145 cells stably expressing WT-AR-K618, or mutant AR-618R, or AR-618Q, respectively (Figure 3A). Cells expressing AR-618Q proliferated more rapidly, but AR-618R demonstrated significantly decreased proliferation as compared to AR-WT (Figure 3B). Consistently, cells expressing AR-618Q or WT-AR-K618 grew larger, more abundant colonies while AR-618R showed decreased anchorage-independent growth (Figure 3C). Moreover, cells expressing AR-618Q slightly enhanced xenograft tumor formation in male nude mice as compared with WT-AR-K618, while AR-618R severely inhibited xenograft tumor growth (Figure 3D). At the endpoint of this study, the volume of xenograft tumors expressing AR-618R was only one quarter of those expressing either AR-WT or AR-618Q, and the initial growth point of tumors expressing AR-618R was delayed by four weeks. Together, these data suggest that ARD1-mediated AR acetylation at K618 plays a critical role in prostate tumor growth.

### Acetylated AR is rapidly shuttled into the nucleus

**Figure 4:**
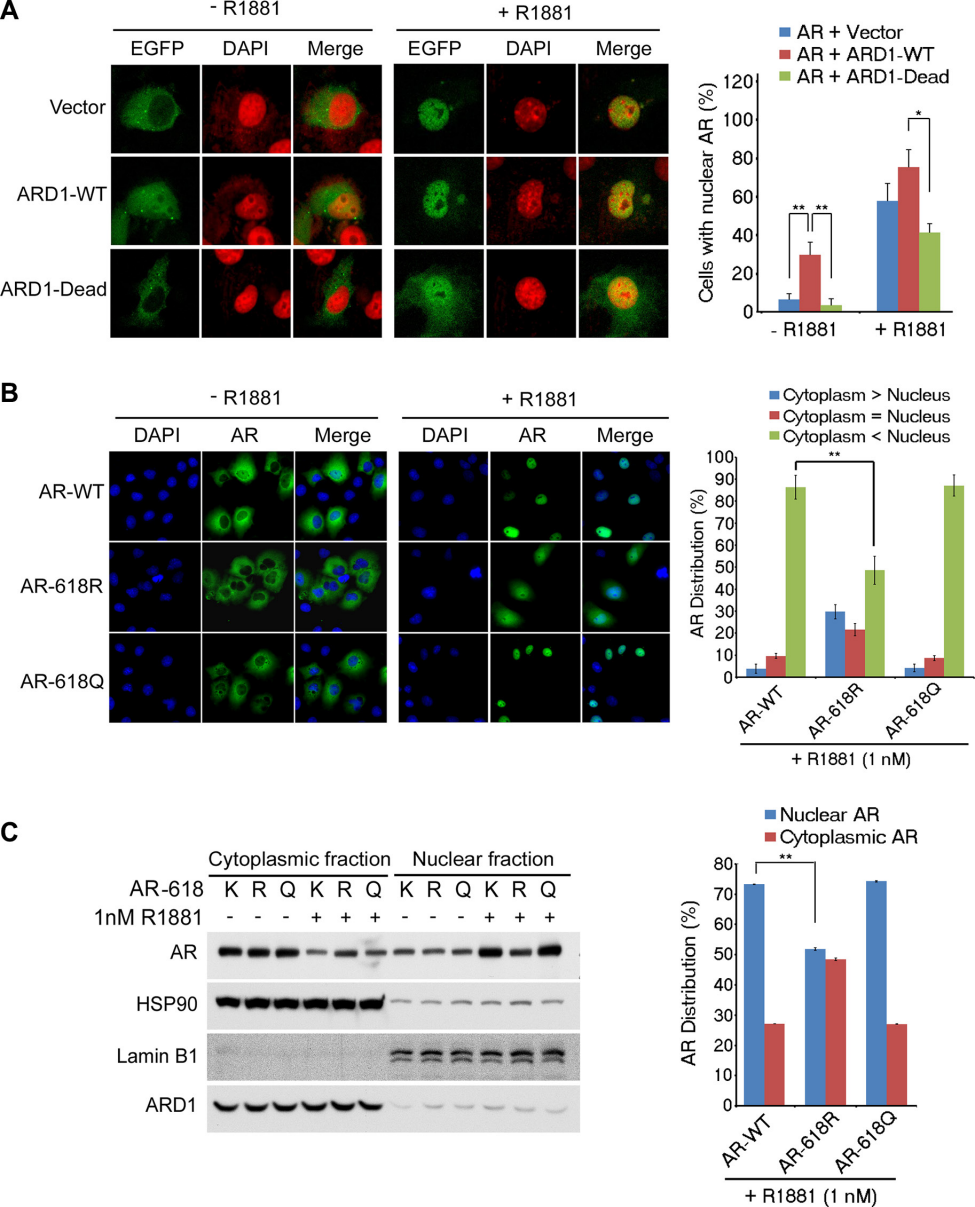
ARD1-mediated AR acetylation at K618 enhances nuclear localization (**A**) AR nuclear localization was analyzed in Cos-7 cells co-transfected with GFP-tagged AR and ARD1-WT, ARD1-dead, or vector in the presence or absence of R1881 by immunofluorescence and confocal microscopy. Proteins indicated include AR (green) or nuclear Dapi (red) stain (left). The percentage of cells with nuclear AR is represented graphically (right) as a mean of three individual fields analyzed ± SD. (**B**) Cos-7 cells were transfected with either AR-WT, AR-618R, or AR-618Q. After 24 hours serum-starvation, cells were treated with ethanol or 1 nM R1881 for one hour. Nuclear localization was analyzed by immunofluorescence confocal microscopy with anti-AR antibody (green) and Dapi staining (blue). Levels of nuclear AR are represented graphically as the mean of three independent fields' ± SD (right) (**C**) Immunoblot of nuclear and cytoplasmic fractionation of AR-WT, AR-618R, or AR-618Q in Cos-7 cells described above in the presence or absence of R1881 with normalized relative densitometry (left). Nuclear versus cytoplasmic levels of AR-WT, AR-618R, or AR-618Q in the presence of R1881 are represented graphically as the mean of three individual replicates ± SD (right).

Increased AR activity and RNA Pol II recruitment following AR acetylation at K618 suggest that AR acetylation by ARD1 may facilitate AR nuclear translocation (Figure 2). To test this hypothesis, we co-transfected WT-ARD1 and GFP-tagged AR into Cos-7 cells and observed that AR readily translocated into the nucleus (Figure 4A). In contrast, co-transfection of ARD1-acetyltransferase-dead and AR displayed limited AR nuclear translocation, suggesting that ARD1-mediated acetylation may lead to AR nuclear entry. Consistently, when we transfected AR-WT, -618R, or -618Q into Cos-7 cells, respectively, and subjected each cell group to one hour of androgen exposure, AR-WT and AR-618Q rapidly entered the nucleus while AR-618R demonstrated significantly reduced ligand-dependent nuclear translocation (Figure 4B). Western blot analysis confirmed less AR-618R present in the nuclear fraction and much more present in the cytoplasmic fraction as compared to the level of AR-WT or AR-618Q post ligand exposure (Figure 4C). These data indicate that ARD1-mediated AR acetylation at K618 enhances ligand-induced AR nuclear translocation.

### Acetylation of AR at K618 promotes androgen-induced AR-HSP90 dissociation

**Figure 5:**
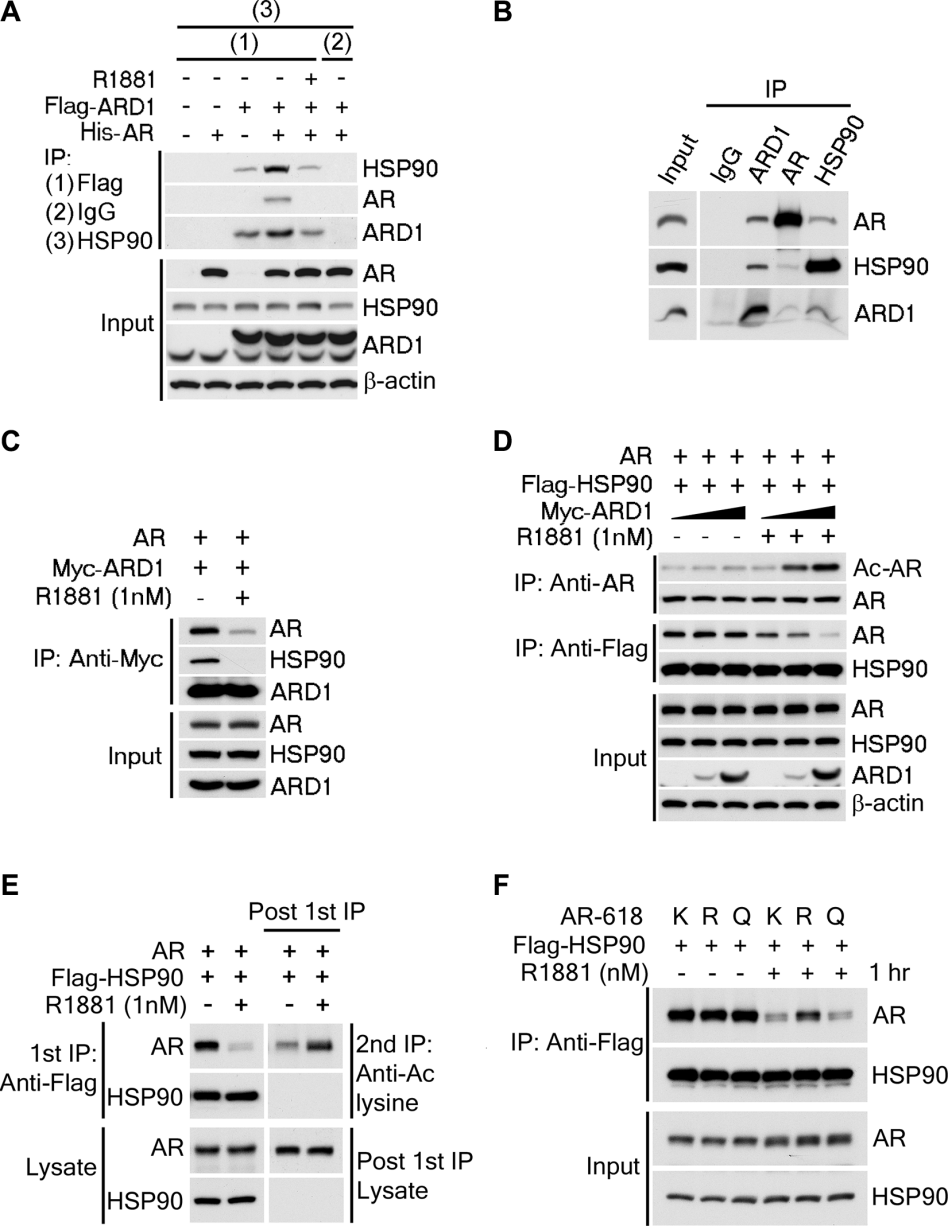
ARD1-mediated AR acetylation drives ligand-induced AR-HSP90 dissociation (**A**) His-AR and Flag-ARD1 were co-transfected in 293T cells. *In vitro* interaction of AR, ARD1, and HSP90 were analyzed by sequential immunoprecipitation and immunoblotting with antibodies as indicated. (**B**) *In vivo* interaction of endogenous ARD1, AR, and HSP90 in LNCaP cells by immunoprecipitation and immunoblotting using antibodies as indicated. (**C**) Immunoblot of interaction between AR-WT and Myc-ARD1-WT transfected in Cos-7 cells in the presence or absence of R1881, immunoprecipitated with anti-Myc antibody. (**D**) AR and Flag-HSP90 were co-transfected with increased amounts of Myc-ARD1 in Cos-7 cells. Ligand-induced and ARD1 dose dependent AR acetylation were measured by immunoprecipitation with an anti-AR antibody and immunoblotted with an antibody specifically against acetylated lysine, while AR-HSP90 dissociation was measured by immunoprecipitation with an anti-Flag antibody and HSP90 co-immunoprecipitated AR was observed by Western blot. (**E**) AR and Flag-HSP90 co-transfected in Cos-7 cells in the presence or absence of R1881 were immunoprecipitated (first IP) with an anti-Flag antibody and immunoblotted using an antibody against AR (left two lanes). A secondary immunoprecipitation was performed on the supernatant after first IP using antibody specifically against acetylated lysine and immunoblotted with an anti-AR antibody (right two lanes). (**F**) Flag-HSP90 co-transfected with AR-WT, or -618R, or -618Q into Cos-7 cells, respectively. Post-transfection, cells were exposed to ethanol or R1881 for one hour. HSP90-bound AR was measured by immunoprecipitation with an anti-Flag antibody and immunoblotted with an anti-AR antibody.

To determine the mechanism through which ARD1-mediated AR-acetylation promotes AR activation and nuclear translocation, we first performed sequential coimmunoprecipitation analyses of ectopically expressed ARD1 and AR proteins in 293T cells and showed that ARD1, AR, and HSP90 form a complex *in vitro* (Figure 5A). Consistently, reciprocal coimmunoprecipitation analysis of the three proteins in LNCaP cells demonstrated that they also form a complex *in vivo* (Figure 5B). Intriguingly, following exposure to ligand, the affinities of the three proteins in the complex were greatly reduced resulting in dissociation of AR and HSP90 from ARD1 (Figure 5C). Since androgen not only induces ARD1-mediated AR acetylation but also AR-HSP90 dissociation [7, 22], we next investigated whether ligand-induced AR acetylation by ARD1 regulates AR-HSP90 dissociation. We ectopically expressed AR and Flag-HSP90 proteins with an increased amount of Myc-ARD1 protein in LNCaP cells. We found that without ligand induction, increased expression of ARD1 does not change the amount of HSP90-bound AR that is immunoprecipitated with HSP90 nor the levels of AR acetylation detected by acetylation-specific antibody (Figure 5D; left 3 lanes). However, following ligand induction, increased expression of ARD1 significantly increases the level of AR acetylation but reduces the level of HSP90-bound AR (Figure 5D; right 3 lanes). These data indicate that ligand-induced AR-HSP90 dissociation correlates with increased AR acetylation in an ARD1 dose dependent manner.

The requirement of ARD1-mediated AR acetylation for ligand-induced AR-HSP90 dissociation was further analyzed. Sequential immunoprecipitation analysis was employed to study the acetylation levels of HSP90-associated- and dissociated-AR following ligand induction. As expected, the acetylation level of HSP90-bound AR immunoprecipitated with HSP90 was dramatically reduced following ligand exposure (Figure 5F; left 2 lanes). However, after a second immunoprecipitation with an acetylation-specific antibody in the residual cell supernatant depleted of HSP90-AR complexes, the HSP90-unbound AR was found to be highly acetylated (Figure 5F; right 2 lanes), suggesting that only acetylated AR dissociates from HSP90 complex following ligand induction.

Finally, we investigated if ARD1-mediated acetylation at AR K618 is responsible for ligand-induced AR-HSP90 dissociation. When HSP90 was co-transfected with AR-K618, AR-618R, or AR-618Q, respectively, we observed that without ligand induction, the interaction levels between HSP90 with AR-K618, AR-618R or 618Q mutants were very similar (Figure 5E; left 3 lanes). But, following ligand induction, the levels of dissociation between HSP90 and the AR-K618 or the acetylation mimetic AR-618Q mutant were greatly reduced, however, the level of the acetylation defective AR-618R mutant was not (Figure 5E; left 3 lanes). From these data, we conclude that ARD1-mediated AR acetylation at K618 is required for ligand-induced AR-HSP90 dissociation, which is probably necessary for AR nuclear translocation, AR target gene expression, and prostate tumorigenesis.

## DISCUSSION

**Figure 6:**
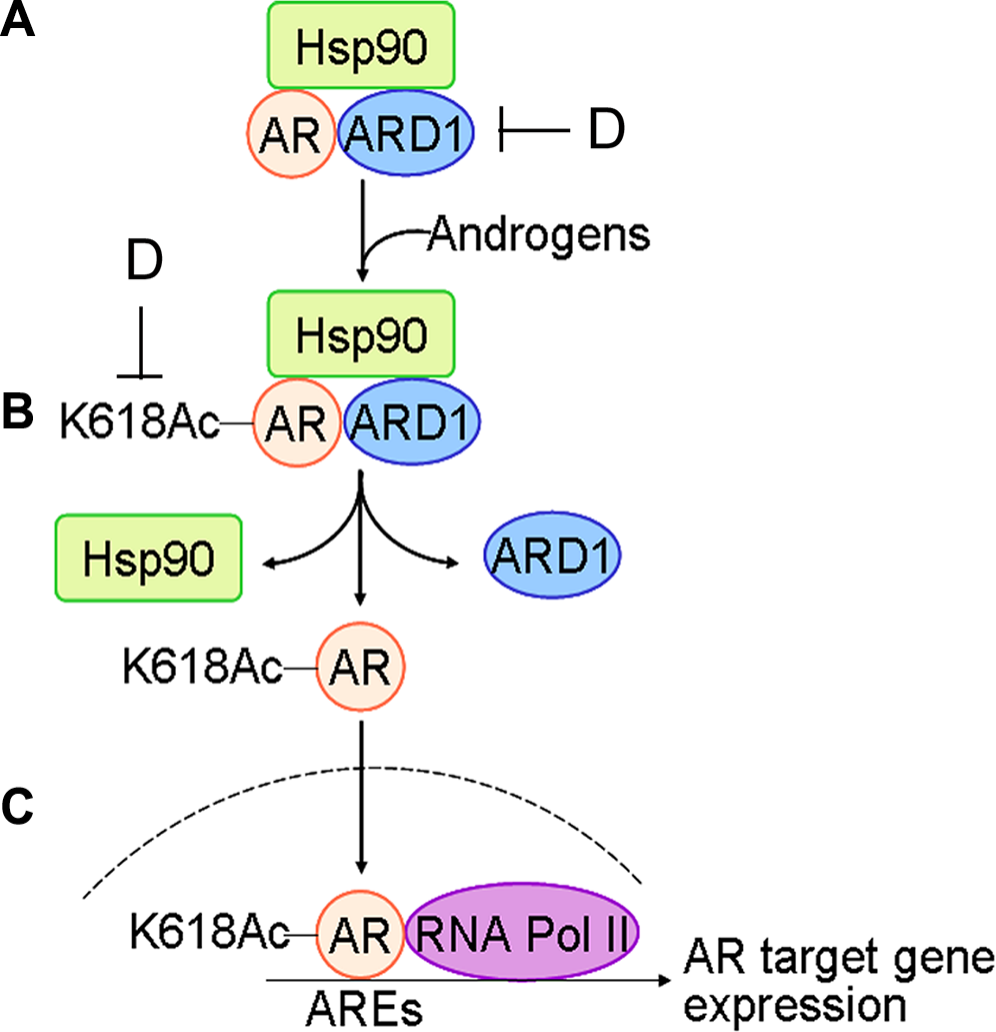
Schematic for the mechanism of ARD1-mediated AR acetylation in prostate tumorigenesis: (A) with limited androgen, ARD1, AR, and HSP90 form a complex in prostate cells (**B**) Following exposure to androgen, ARD1 acetylates AR resulting in dissociation of acetylated AR from HSP90 and ARD1. (**C**) Acetylated AR is translocated into nuclei and binds to ARE and RNA Pol II for AR target gene expression and prostate tumorigenesis. (**D**) Inhibition of ARD1 or ARD1-mediated AR acetylation may prevent AR nuclear entry and androgen-dependent prostate tumorigenesis.

Androgen-induced AR dissociation from a complex with HSP90 is the first step in AR activation. However, the molecular mechanisms involved in this event remain incompletely understood. In the present study, we uncovered a new acetylation site in AR and demonstrated that ARD1-mediated AR acetylation at K618 is required for ligand-induced AR-HSP90 dissociation, AR activation, and prostate tumorigenesis, and suggested a novel ARD1-AR-HSP90 axis for therapeutic intervention (Figure 6). In addition, we provided evidence that AR-acetylation at K618 increases AR nuclear translocation (Figure 4). It has been reported that ligand-induced AR nuclear translocation can be mediated by Importin-7 or Hsp27 [23, 24]. However, our experiments demonstrated that the mutant AR-618R does not affect AR-Importin-7 or AR-Hsp27 interaction in LNCaP cells following ligand exposure, suggesting that the process of ARD1-mediated nuclear translocation seems to be Importin-7 or Hsp27-independent (Figure S3, S4). Further analysis will be required to understand the mechanisms by which ligand-induced AR-K618 acetylation by ARD1 facilitates AR nuclear translocation in prostate tumorigenesis.

It is also worth noting that independent of K618, AR K605 seems to also be acetylated by ARD1 relative to the DBD-Total mutant (Figure 1C), though at a significantly lower level than K618. We examined the capacity of constitutive acetylation at K605 to induce PSA-luc reporter activity and found that there was no statistical difference between AR-WT and AR-605Q (data not shown). This result indicates that low levels of acetylation at K605 may not play a meaningful role in ARD1-mediated AR activation.

The current rationale for HSP90 inhibitors is based on blocking HSP90 ATPase activity with a nucleotide mimetic, driving client protein proteolysis. To this date, this strategy has demonstrated a limited clinical success in the treatment of PCa [10, 25]. This result is likely due in part to HSP90 targeting and inhibition of other client proteins such as c-Src and ErbB-2 [26, 27]. Our finding that ARD1-mediated AR acetylation at K618 activates AR and induces ligand-dependent AR-HSP90 dissociation offers a mechanism by which AR activation can be interrupted independent of HSP90 inactivation.

In conclusion, we have identified a novel post-translational activating modification of AR that promotes AR-HSP90 dissociation, AR target gene expression, and PCa cell growth. Targeting ARD1-mediated AR acetylation and AR-HSP90 dissociation without disrupting the critical functions of HSP90 may prove to be a potent alternative to HSP90 inhibition while simultaneously avoiding the deleterious effects of androgen deprivation therapy.

## MATERIALS AND METHODS

### Cell lines

HEK293T, DU 145, LNCaP, and Cos-7 cells were purchased from the American Type Culture Collection (ATCC). Cells were maintained in appropriate media per the ATCC guidelines. Where applicable, cells were treated with deacetylation inhibition cocktail (sc-362323) diluted in DMSO.

### Immunoblotting and immunoprecipitation

Immunoblotting and immunoprecipitation were performed as previously described [28]. The primary antibodies utilized in this study include androgen receptor (AR) (sc-7305 and sc-815), ARD1 (sc-33820), acetylated lysine (sc-32268), mouse IgG (sc-2025), and His (sc-804) (Santa Cruz Biotechnology); β-actin (A5441) and FLAG (F3165) (Sigma); RNA polymerase II (AbCam 5408); HSP90 (Thermo Pierce PA3-013); GST (Chemicon AB3282). HRP-conjugated secondary antibodies used were from Sigma. Immunoprecipitation was performed using lysis/wash buffer [50 mM Tris-HCl (pH = 7.4), 150 mM NaCl, 1 mM EDTA, 1% Triton-X, 1× protease inhibitor cocktail]. Protein G agarose beads (Thermo 20399) were used to immunoprecipitate target proteins.

### Plasmids and transient or stable transfection

The FLAG-ARD1 construct was generated as previously described [22]. The PSA-luciferase (PSA-luc) reporter was kindly provided by Dr. Haojie Huang (Mayo Clinic). The pLenti-AR construct was generously provided by Dr. Yan Dong (Tulane University), and subsequent mutant AR and ARD1-dead constructs were generated using Quikchange XL site-directed mutagenesis kit (Life Technologies). Transient transfections of plasmids were performed using Transit-X2 transfection reagent (Mirus) according to the manufacturer's recommendations. Stable DU 145 cells expressing WT or mutant AR were generated using Virapower lentiviral packaging kit (Invitrogen) according to the manufacturer's recommendations.

### *In vitro* and *in vivo* acetylation assay

The *in vitro* acetylation assay on AR was performed using His-ARD1 as described previously with modification [22]. Briefly, GST-tagged AR fragments were grown overnight in BL21 *E. coli* cells, induced by .1 mM IPTG for 2 hours. GST-AR fragments were purified using glutathione beads (GE Healthcare 17-0756-01), eluted with reduced glutathione, and dialyzed for 24 hours using Slide-A-Lyzer .5–3 ml dialysis cassettes (Thermo 66380). His-ARD1 was purified using a His SpinTrap Kit (GE Healthcare 28-4013-53) per the manufacturer's recommendations. The *in vitro* acetylation assay was performed and proteins were separated by SDS-PAGE. Corresponding amounts of each fragment were separated by SDS-PAGE and stained with Coomassie blue stain. Cell proliferation and anchorage-independent growth assays. Cell proliferation MTT assays were performed with DU 145 cell pools stably expressing WT or mutant AR using CellTiter 96 Non-Radioactive Cell Proliferation Assay (Promega) kit according to the manufacturer's recommendations. Colonogenic growth of stable cells was analyzed by evaluation of growth in soft agar 17 days post-plating, as described previously [29].

### Xenograft tumor growth

Stable DU 145 cell pools expressing WT or mutant AR (*n* = 3 mice per group. 2.5 × 10^6^ cells/injection) were injected into the hind flanks of nude mice (Charles River) as previously described [22]. Growth was monitored by caliper measurement over the course of 10 weeks. All procedures follow an animal use protocol approved by the Institutional Animal Care and Use Committee of Louisiana State University Health Sciences Center in New Orleans.

### Luciferase reporter assays

DU 145 cells were transfected with a combination of plasmids, including either PSA-Luc, as well as a vector containing either WT or 618K-mutant AR, and an internal control (pRL-TK), and serum-starved for 24 hours. Cells were then subjected to 24 hours of R1881 exposure. Analysis was performed using the Dual-Luciferase Reporter Assay system (Promega) per the manufacturer's instructions.

### Quantitative RT-PCR

Total RNAs were prepared using the RNeasy kit (Qiagen) and cDNA was synthesized using Superscript III First-Strand synthesis system (Invitrogen). Quantitative RT-PCR (qRT-PCR) was performed using SYBR Green detection method on a CFX96 Real-Time PCR detection system (BIO-Rad). All of the primers used for this study have been reported [22].

### Chromatin immunoprecipitation

ChIP assays were performed using SimpleChIP Plus Enzymatic Chromatin IP kit (Cell Signaling) as previously reported with a few modifications [30]. Briefly, DU 145 cells were transiently transfected with vectors either expressing WT or K618-mutantAR. PCR was performed with previously reported primers [22], and the relative enrichment was demonstrated using agarose gel electrophoresis.

### Nuclear entry assay

Cos-7 cells were plated on chamber slides in phenol red-free RPMI 1640 media with 10% charcoal-depleted FBS. Cells were transfected after 24 hours with either AR-WT, AR-618R, or AR-618Q. Cells were treated with 1 nM R1881 for one hour 24 hours post-transfection. Cells were embedded on slides with paraformaldehyde, permealized with .25% triton-x, blocked with 5% goat serum, and probed with anti-AR antibody (sc-7305) overnight. Anti-mouse secondary antibody (Alexa Fluor 448) was applied, as well as Hoechst stain (Thermo 33342) specific for DNA.

### Statistical analysis

Data are expressed as mean ± SD from at least 3 experiments. Statistical analyses were performed by Student's *t* test. *P* < 0.05 was considered statistically significant.

## SUPPLEMENTARY MATERIALS FIGURES


