# Supplementary material for: Acetylation of androgen receptor by ARD1 promotes dissociation from HSP90 complex and prostate tumorigenesis
Source: Oncotarget. 2016 Sep 21;7(44):71417–28. doi: 10.18632/oncotarget.12163 (PMC5342088; doi:10.18632/oncotarget.12163)
Supplement: Supplementary file 1 [file oncotarget-07-71417-s001.pdf]

Acetylation of androgen receptor by ARD1 promotes dissociation from HSP90 complex and prostate tumorigenesis

Supplementary Materials

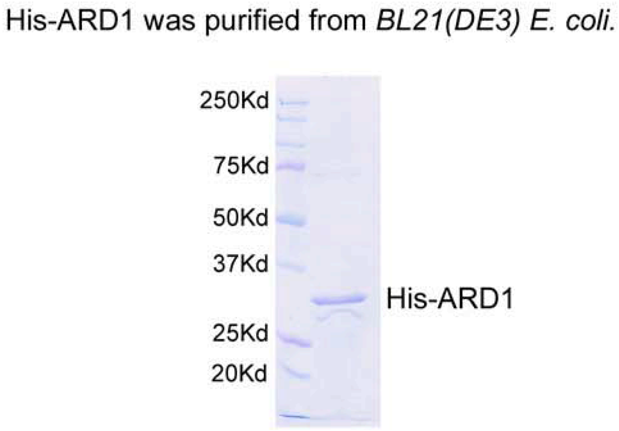

Supplementary Figure S1: Purified WT-ARD1 used in *in vitro* acetylation assays. His-tagged WT-ARD1 was grown up in BL21 (DE3) *E. coli*, purified with His SpinTrap Kit (GE Healthcare Life Sciences) and stained with Coomassie blue.

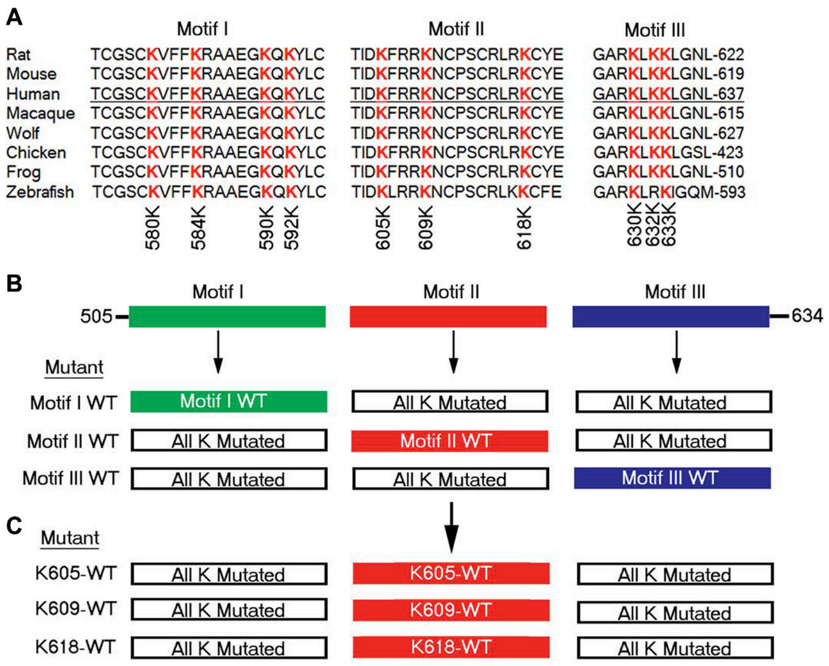

Supplementary Figure S2: Schematic and amino acid sequences of AR DBD mutants used in *in vitro* acetylation assays. (**A**) Amino acid sequences of the three lysine-containing motifs within AR DNA-binding domain (DBD) demonstrating cross-species conservation. (**B**) Schematic of motif serial mutations in which the lysines within the colored motifs were retained while the lysines in open motifs were all mutated. (**C**) Individual lysine serial mutations within the DBD.

AR-K618 acetylation does not impair IPO7/AR interaction

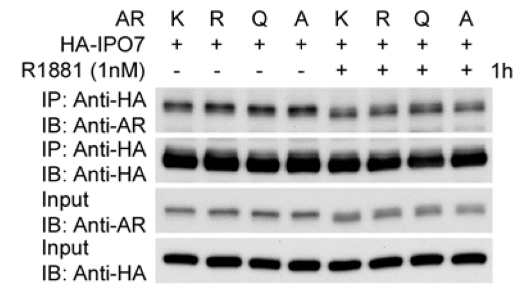

Supplementary Figure S3: Acetylation at AR-K618 does not impair Importin-7 (IPO7)-AR interaction. AR and HA-IPO7 were co-transfected into Cos-7 cells after cultured in androgen-free medium for 48 hours. 24 hours after transfection, cells were subjected to one hour of either ethanol or R1881 exposure. AR-IPO7 interaction was detected by immunoprecipitation and immunoblotting with antibodies as indicated.

AR-K618 acetylation does not affect AR and Hsp27 interaction

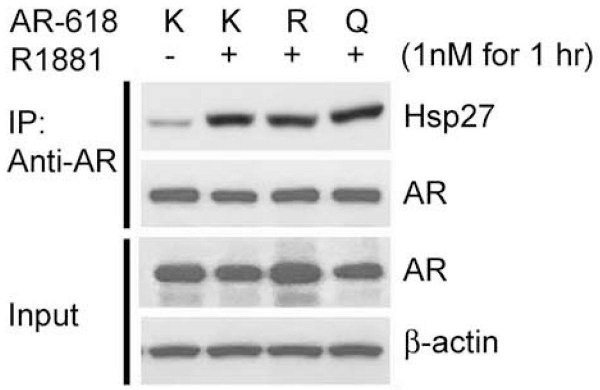

Supplementary Figure S4: Acetylation at AR-K618 does not affect AR-Hsp27 interaction. Wild-type AR-618K (K), mutant AR-618R (R), or AR-618Q construct was transiently transfected into LNCaP cells, respectively, and maintained in medium with 1nM R1881 for 1 hour. The cell lysates were then immunoprecipitated using AR antibody and the level of Hsp27 interacted with AR was measured by Western blot using Hsp27 antibody (Enzo Life Sciences).
